# Efficient plant regeneration from embryogenic cell suspension cultures of *Euonymus alatus*

**DOI:** 10.1038/s41598-021-94597-4

**Published:** 2021-07-23

**Authors:** Hyun-A Woo, Seong Sub Ku, Eun Yee Jie, HyeRan Kim, Hyun-Soon Kim, Hye Sun Cho, Won-Joong Jeong, Sang Un Park, Sung Ran Min, Suk Weon Kim

**Affiliations:** 1 Plant Systems Engineering Research Center, Korea Research Institute of Bioscience and Biotechnology, 125 Gwahak-ro, Yuseong-gu, Daejeon, 34141 Republic of Korea; 2 Biological Resource Center, Korea Research Institute of Bioscience and Biotechnology, 181 Ipsingil, Jeongeup‑si, Jeollabuk‑do 56212 Republic of Korea; 3 Department of Crop Science, Chungnam National University, 99 Daehak-ro, Yuseong-gu, Daejeon, 34134 Republic of Korea

**Keywords:** Biological techniques, Biotechnology, Developmental biology, Plant sciences

## Abstract

To establish an efficient plant regeneration system from cell suspension cultures of *Euonymus alatus*, embryogenic callus formation from immature embryos was investigated. The highest frequency of embryogenic callus formation reached 50% when the immature zygotic embryos were incubated on Murashige and Skoog (MS) medium supplemented with 1 mg/L 2,4-dichlorophenoxy acetic acid (2,4-D). At higher concentrations of 2,4-D (over 2 mg/L), the frequency of embryogenic callus formation declined significantly. The total number of somatic embryos development was highest with the 3% (w/v) sucrose treatment, which was found to be the optimal concentration for somatic embryo formation. Activated charcoal (AC) and 6-benzyladenine (BA) significantly increased the frequency of plantlet conversion from somatic embryos, but gibberellic acid (GA_3_) had a negative effect on plantlet conversion and subsequent development from somatic embryos. Even though the cell suspension cultures were maintained for more than 1 year, cell aggregates from embryogenic cell suspension cultures were successfully converted into normal somatic embryos with two cotyledons. To our knowledge, this is the first successful report of a plant regeneration system of *E. alatus* via somatic embryogenesis. Thus, the embryogenic cell line and plant regeneration system established in this study can be applied to mass proliferation and production of pharmaceutical metabolite in *E. alatus*.

## Introduction

*Euonymus alatus* is a deciduous shrub belonging to the family Celastraceae that is mostly native to East Asia. It can grow well in a wide range of soils and environmental conditions^1^. Because of its proliferation characteristics with prodigious seed production and dispersal, it is considered one of the most invasive ornamental shrubs by American gardeners^2^. Thus, the development of sterile, noninvasive, seedless triploid cultivars using endosperm cultures of *E. alatus* is being pursued^1^.

The seeds of *E. alatus* produce high levels of unusual 1,2-diacyl-3-acetyl-sn-glycerols (acTAGs), where the *sn*-3 position is esterified with acetate instead of the long-chain fatty acid as their major storage lipids^3,4^. The *sn*-3 acetyl group imparts acTAGs with different physical and chemical properties, by reducing their viscosity by approximately 30% compared to that of regular triacylglycerols^4^. The reduced viscosity of acTAGs is a significantly important characteristic in the production of biodiesel and lubricants as well as in food production and human nutrition^5^. Genes encoding DGATs from *Euonymus* species could provide insights for the biotechnological production of acTAGs in plant cell culture systems and transgenic plants. Thus, *Euonymus* plant species are considered a useful biological resource of unique genes modifying the content and composition of vegetable oils^6^.

In general, *Euonymus* is traditionally used as a medicinal plant in many Asian countries. More than 230 chemical compounds have been identified and isolated from it, including sesquiterpenoids, diterpenoids, triterpenoids, flavonoids, phenylpropanoids, lignans, steroids, alkaloids, and other compounds^7,8^. Thus, *Euonymus* plant species have potential in the treatment of injury and inflammation^9,10^ as well as diseases, including cancer, diabetes^11^, and hyperglycemia^12,13^. Due to the medicinal importance of *E. alatus* plants, many studies have tried to identify the chemical compounds corresponding to their pharmacological effects^8^.

Plant cell and tissue culture can be utilized as a new means of producing high-value recombinant proteins or pharmaceutical metabolites^14–16^. To achieve this goal, a plant cell culture system, an efficient plant regeneration system via organogenesis and somatic embryogenesis, and in vitro mass proliferation system must be established. However, there have been only a few studies on the in vitro cultivation of *E. alatus* to date. A few studies have reported plant regeneration through adventitious shoot formation. Smith and Jernstedt^17^ reported in vitro plant regeneration via adventitious shoot formation from hypocotyl explants, but the efficiencies of both shoot and root formation were relatively low. To develop sterile, seedless triploid plants, a plant regeneration system was also established from immature and mature endosperm explants. Triploid plant regeneration rates were 0.42% from immature endosperm explants and 0.34% from mature endosperm explants, respectively^1^. Moreover, Chen et al*.*^2^ reported the *Agrobacterium*-mediated genetic transformation protocol of *E. alatus* using kanamycin as a selection agent. However, despite the pharmacologically very important plant resource of *E. alatus*, there have been no reports of plant regeneration studies from cell suspension cultures via somatic embryogenesis to date.

Therefore, this study attempted to establish an efficient plant regeneration system through somatic embryogenesis of *E. alatus*. First, the effect of 2,4-D concentrations on the formation of embryogenic calli from immature zygotic embryos was examined. Second, the effects of sucrose concentrations, growth regulators (BA and GA_3_), and AC in the culture medium on somatic embryogenesis and germination from somatic embryos to establish an efficient plant regeneration system were also examined. Finally, embryogenic cell suspension cultures from embryogenic calli were developed.

## Results

### Effect of 2,4-D on embryogenic callus formation

**Figure 1:**
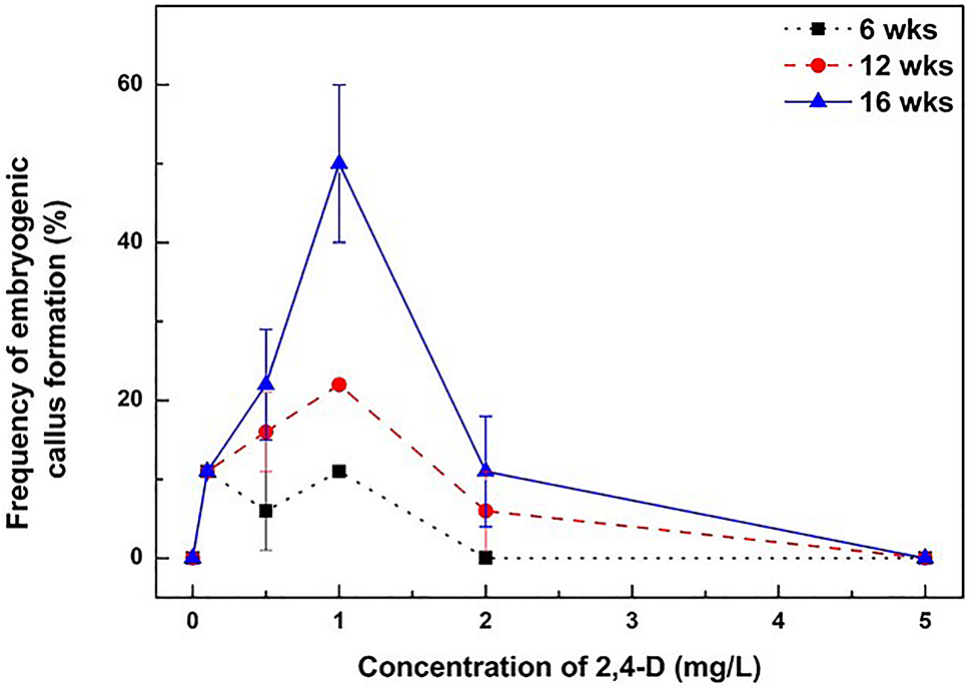
Effects of 2,4-D concentrations on embryogenic callus formation from immature zygotic embryos. Each symbol () represent the incubation periods of immature embryos cultured on MS medium supplemented with several concentrations of 2,4-D. The frequency of embryogenic callus formation from different concentrations of 2,4-D treatments. Each treatment consisted of 9 immature zygotic embryos in a Petri dish and was repeated three times. Error bars represent SE (N = 27).

The effect of 2,4-D on embryogenic callus formation from immature embryo cultures was examined (Fig. 1). After 6 to 16 weeks of culture, the highest frequency of embryogenic callus formation reached 50% when the immature zygotic embryos were incubated on MS medium supplemented with 1 mg/L 2,4-D. Under a lower concentration of 2,4-D (less than 1 mg/L 2,4-D), the frequency of embryogenic callus formation from immature embryo cultures was increased in a dose-dependent manner. However, the frequency of embryogenic callus formation declined significantly under the higher concentration of 2,4-D treatment (over 2 mg/L 2,4-D). Interestingly, the frequency of embryogenic callus formation increased by approximately 2 times under the 0.5–2 mg/L 2,4-D treatments as the incubation period was extended. However, immature embryos did not form any embryogenic calli at all in the 5 mg/L 2,4-D treatments, even though the incubation period was prolonged. These results clearly showed that 1 mg/L 2,4-D is the most suitable concentration for embryogenic callus formation from immature embryos.

**Figure 2:**
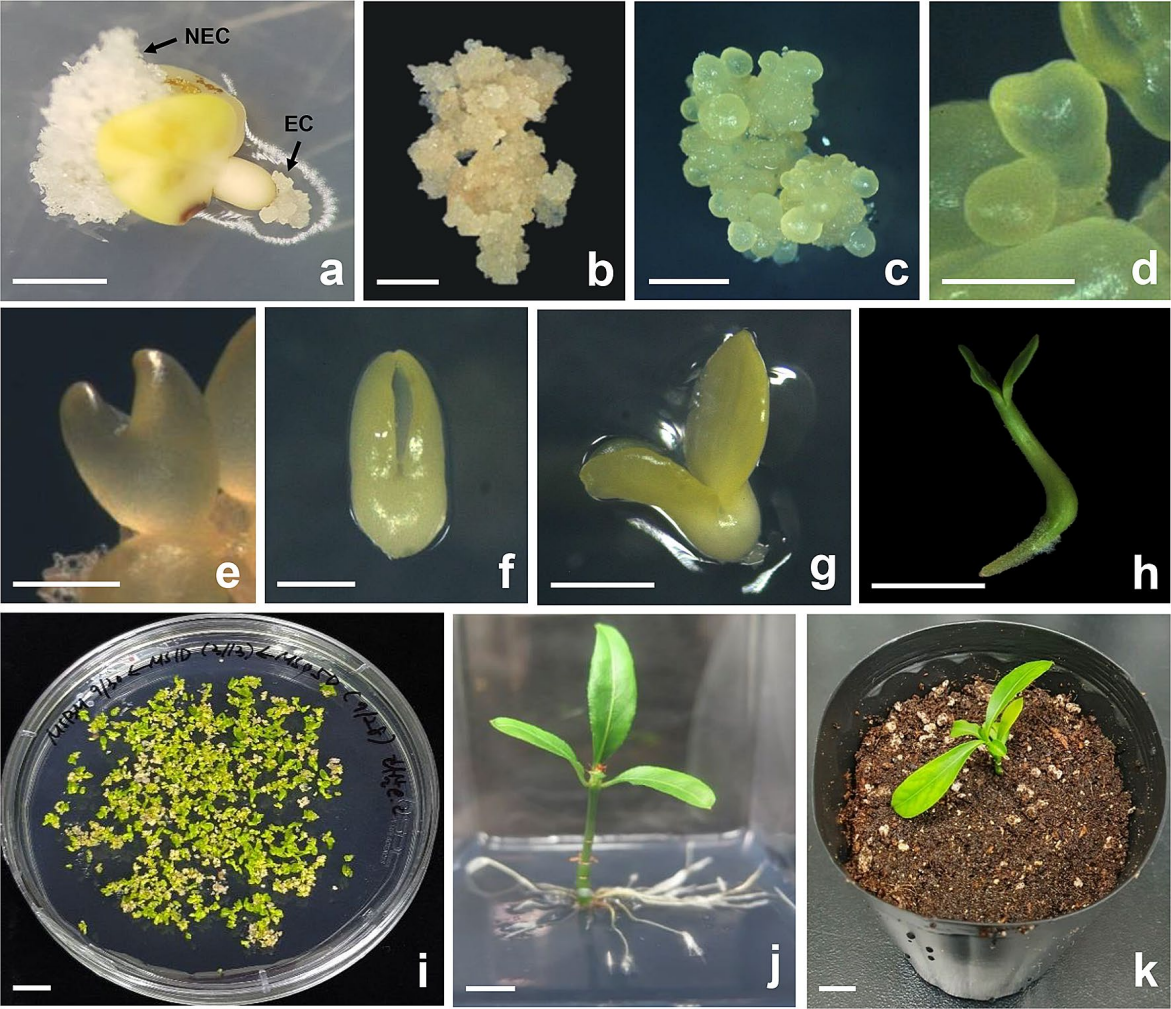
Somatic embryogenesis and plant regeneration from immature zygotic embryo cultures of *E. alatus*. (**a**) Embryogenic and nonembryogenic callus formation from zygotic embryo; (**b**) proliferation of embryogenic callus; (**c**) numerous globular-shaped somatic embryos formation from embryogenic callus; (**d**,**e**) heart-shaped somatic embryo; (**f**) torpedo-shaped somatic embryo; (**g**) cotyledonary somatic embryo; (**h**) germination of somatic embryo; (**i**) development of numerous somatic embryos from embryogenic calluses cultured on MS basal medium; (**j**) plantlet developed from somatic embryo; (**k**) regenerated plant transplanted to potting soil. Arrows represent embryogenic (EC) and nonembryogenic callus (NEC). Scale bars represent 1 mm (**a**–**c**,**g**), 0.5 mm (**d**–**f**), and 1 cm (**h**–**k**), respectively.

An efficient plant regeneration system from embryogenic calli was established in this study (Fig. 2). Two different types of calli were formed simultaneously from immature embryos when they were cultured on MS medium supplemented with 1 mg/L 2,4-D. White, spongy-like calli were formed in the cotyledons of immature embryos after 4 weeks of incubation, whereas white nodular calli were mainly formed at the root regions of immature embryos, including the root tips, after 6 weeks of incubation (Fig. 2a). When the white nodular calli were carefully transferred to fresh MS medium supplemented with 1 mg/L 2,4-D, these calli easily proliferated (Fig. 2b). These embryogenic calli were subcultured on fresh medium every 4 weeks. During callus proliferation, white nodular calli transferred to fresh MS basal solid medium developed into many light yellow globular stage somatic embryos (Fig. 2c) after 4 weeks of culture. Globular stage somatic embryos further developed into heart (Fig. 2d,e), torpedo (Fig. 2f), and cotyledonary stage embryos (Fig. 2g). After an additional 4 weeks of incubation in the light, somatic embryos successfully germinated (Fig. 2h) and developed into normal plantlets (Fig. 2j). The normal plantlets were transplanted into potting soil (Fig. 2k) and maintained in a growth chamber. These results clearly indicated that initial white nodular calli from immature embryos were embryogenic, and whole plant regeneration through the somatic embryogenesis pathway could be possible.

### Effect of sucrose and light on the development of somatic embryos

**Figure 3:**
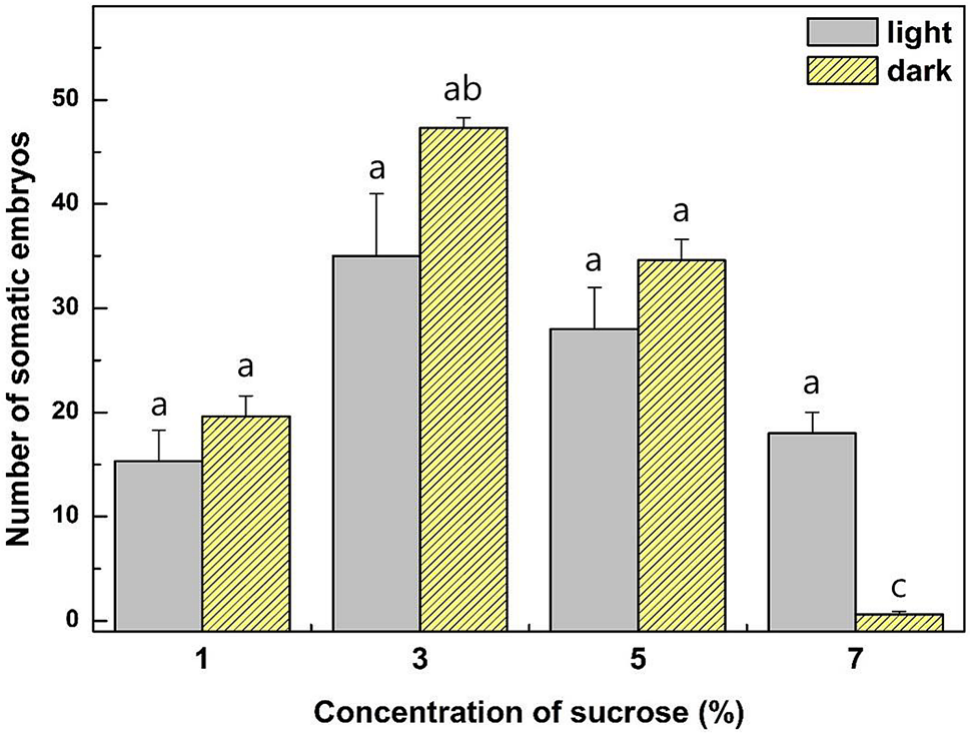
Effects of sucrose concentrations and light requirement on somatic embryo formation from embryogenic callus. The number of somatic embryos from different combinations of treatments of 1, 3, 5, 7% sucrose in the light or dark incubation. Each treatment consisted of 10 embryogenic calli in a Petri dish and was repeated three times. Error bars represent SE (N = 30). Different lowercase letters on the bars indicate significant differences between each treatment at the 5% level (ANOVA followed by a Turkey’s test, p < 0.05).

The effect of sucrose concentration on somatic embryo formation from embryogenic calli was examined (Fig. 3, Supplementary Fig. S1). After 5 weeks of culture, the total numbers of normal somatic embryos with two cotyledons were counted under a dissecting microscope. In the case of dark incubation, the total number of somatic embryos reached 47.3 with the 3% sucrose treatment, which was higher than with the other sucrose concentration treatments (Fig. 3). The total numbers of somatic embryos from embryogenic calli declined as the sucrose concentration increased.

The total numbers of somatic embryos from the 1, 3, 5, and 7% (w/v) sucrose treatments under light incubation were 15.3, 35, 28 and 18, respectively (Fig. 3, Supplementary Fig. S1). In the case of light incubation, the overall change in the total number of somatic embryos by sucrose treatment was similar to that in dark incubation except for the 7% (w/v) sucrose treatments. These results clearly showed that light does not play a stimulatory role in early embryo development from embryogenic calli.

### Effect of growth regulators and activated charcoal on the conversion of somatic embryos into plantlets

**Figure 4:**
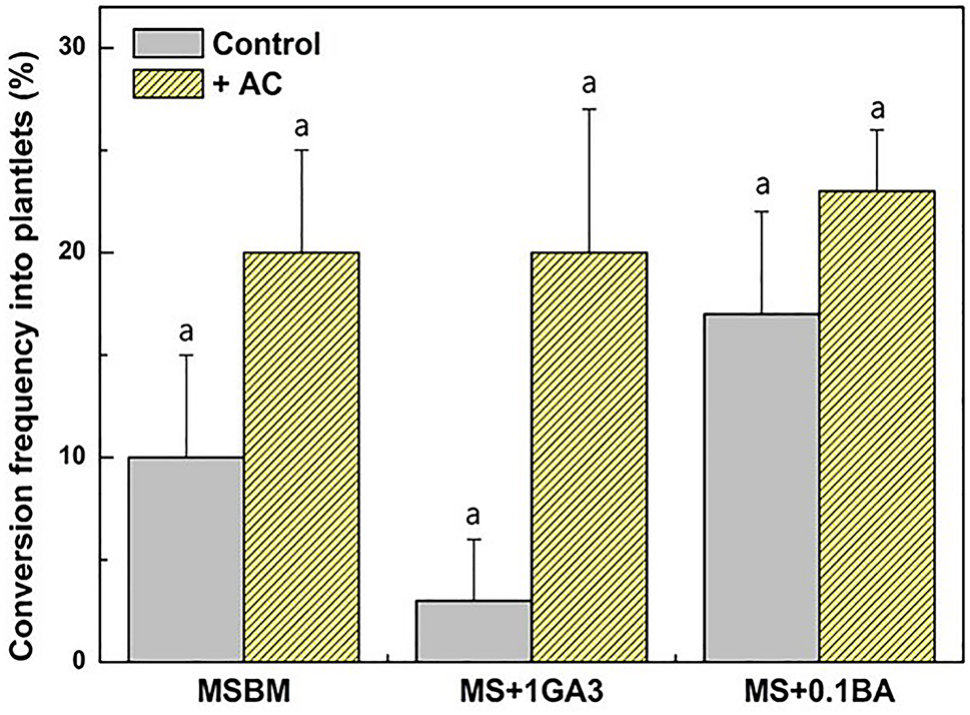
Effects of growth regulators (BA and GA_3_) and activated charcoal (AC) on the conversion of somatic embryos into plantlets. The conversion frequency of somatic embryos for different combinations of treatments of 0.1 mg/L BA, 1 mg/L GA_3_ and 0.2% AC. Each treatment consisted of 10 somatic embryos in a Petri dish and was repeated three times. Error bars represent SE (N = 30). Different lowercase letters on the bars indicate significant differences between each treatment at the 5% level (ANOVA followed by a Turkey’s test, p < 0.05).

To accelerate whole plant regeneration from somatic embryos, the effect of BA and GA_3_ on further conversion of somatic embryos into plantlets was examined. Somatic embryos were transferred to MS medium supplemented with 0.1 mg/L BA or 1 mg/L GA_3_ (Fig. 4, Supplementary Fig. S2). The conversion frequency of somatic embryos into plantlets was 10% when somatic embryos were incubated on MS basal medium. The conversion frequency of somatic embryos from the 0.1 mg/L BA and 1 mg/L GA_3_ treatment was 17% and 3% respectively. The conversion frequency of somatic embryos from the 0.1 mg/L BA treatment was 1.7 times higher than that of the control treatment (Fig. 4). These results clearly showed that the 0.1 mg/L BA treatment had a more stimulating role than the 1 mg/L GA_3_ treatment for the conversion of somatic embryos into normal plantlets.

Furthermore, the effect of AC on the further development of somatic embryos into plantlets was investigated (Fig. 4, Supplementary Fig. S2). The conversion frequency of somatic embryos into plantlets was 20% when somatic embryos were incubated on MS basal medium supplemented with 0.2% AC. The conversion frequency of somatic embryos into plantlets was increased more than 6.7 times under the 1 mg/L GA_3_ + AC treatment compared to that under 1 mg/L GA_3_. Whereas, the conversion frequency under 0.1 mg/L BA + AC treatment was increased 1.4 times compared to that under MS + 0.1 mg/L BA (Fig. 4). These results clearly showed that had a more stimulatory role than plant growth regulator supplementation in the conversion of somatic embryos into plantlets.

### Efficient plant regeneration from embryogenic cell suspension cultures

**Figure 5:**
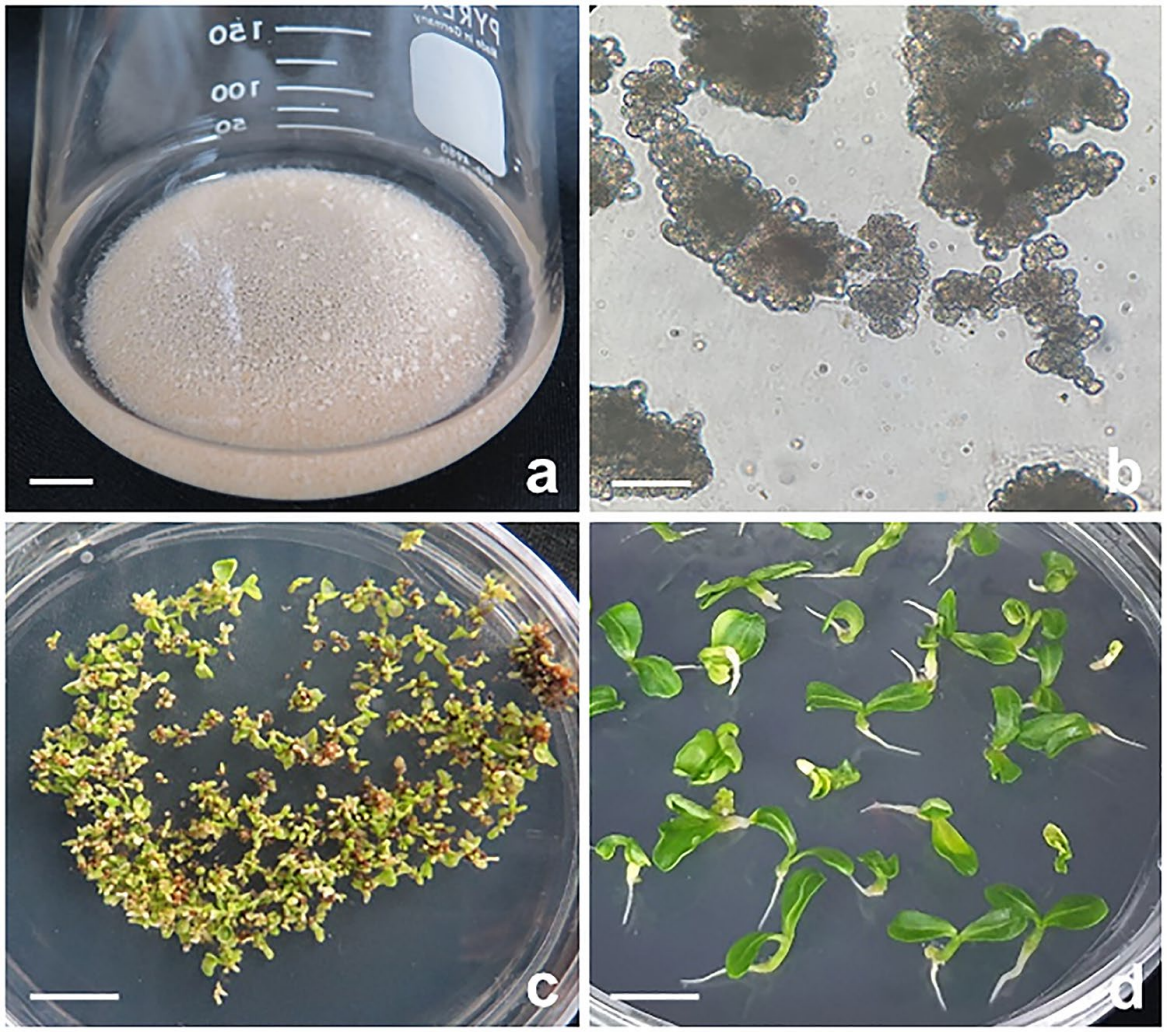
Establishment of plant regeneration system from embryogenic cell suspension cultures. (**a**) Embryogenic cell suspension cultures; (**b**) proliferation of embryogenic cell aggregates; (**c**) development of numerous somatic embryos from cell suspension cultures; (**d**) conversion of somatic embryos into plantlets. Scale bars represent 1 cm (**a**,**c**,**d**), and 200 µm (**b**), respectively.

An embryogenic cell suspension culture was established from immature-derived white calli (Fig. 5). White calli were finely dispersed and proliferated well in MS1D liquid medium. After two rounds of subculture, the cell suspension culture was subcultured at 1-week intervals. The cell suspension cultures showed typical characteristics of embryogenic cells consisting of small isodiametric (approximately 30 µm) cell aggregates with dense cytoplasm (Fig. 5a,b). To investigate whether the cell aggregates have embryogenic potential, cell aggregates from cell suspension cultures were transferred to MS basal medium. After 4 weeks of culture, these cell aggregates were successfully converted into somatic embryos (Fig. 5c). These somatic embryos were able to develop plantlets (Fig. 5d). The embryogenic cell suspension cultures maintained embryogenic potential for more than 1 year. To our knowledge, this is the first successful report of plant regeneration via somatic embryogenesis from cell suspension cultures of *E. alatus*. Thus, the embryogenic cell suspension culture established in this study can be applied in mass proliferation and genetic modification for pharmaceutical metabolite production.

## Discussion

It is well known that auxins, mainly 2,4-D, are required for somatic embryogenesis induction and embryo multiplication in many plant species, including cassava^18^, *Codonopsis lanceolata*^19^, *Opolopanax elatus*^20^, *Kalopanax pictus*^21^, *Schisandra chinensis*^22^. In this study, it was found that the optimum concentration of 2,4-D for the induction of embryogenic calli from immature embryo cultures was 1 mg/L (Fig. 1). This result is similar to the results obtained in *Codonopsis lanceolata*^19^, and *Opolapanax elatus*^20^. Chen et al*.*^22^ reported that a high concentration of 2,4-D was required for the induction of somatic embryogenesis in *Schisandra chinensis*. However, a high concentration of 2,4-D (more than 2 mg/L) inhibited the induction of somatic embryogenesis from immature embryo cultures of *E. alatus*. Our results clearly indicate that even a low concentration of 2,4-D treatment can sufficiently induce embryogenic calli from immature embryos, and whole plant regeneration through the somatic embryogenesis pathway could be possible.

This study describes the first successful plant regeneration from immature embryo cultures via somatic embryogenesis (Fig. 2). Although a few somatic embryos with abnormal cotyledons were formed from embryogenic callus, most normal somatic embryos were successfully developed into plants. In general, somatic embryogenesis plays a significant role in the mass propagation of medicinal plants and has become a routine protocol for many woody plants^23–26^. However, the practical application of somatic embryogenesis in a wide range of woody plants is limited by genotypic influences and poor germination of somatic embryos^24,25^. In general, cold treatment is required for the breaking of seed dormancy to promote seed germination^27^. Moreover, cold storage can promote somatic embryo conversion rates potentially caused by epigenetic changes triggered by temperature stress^23^. In this study, we also examined the effect of cold storage (4 ℃ for 10 days) of immature seeds on somatic embryogenesis. The frequency of embryogenic callus formation from the immature seeds subjected to cold storage was much higher than that of those not subjected to cold treatment. The frequency of embryogenic callus formation from the seeds that did not undergo cold storage was not 2,4-D concentration dependent (Supplementary Fig. S3). Thus, it is inferred that the difference in maturity of zygotic embryos was one of the reasons for the variation in embryogenic callus formation.

In the process of plant regeneration from somatic embryos, it is necessary to remove plant growth regulators at the somatic embryo development and maturation steps. 2,4-D often hampers embryo development and their subsequent conversion into plants at these steps^28,29^. However, over 90% of embryogenic calli gave rise to numerous somatic embryos when transferred to MS basal medium without any growth regulators in this study (Fig. 2i). This result clearly showed that 2,4-D did not have any inhibitory role in the conversion of somatic embryos into plants. Considering these results, we suggest that the plant regeneration system established in this study, combined with genome editing technology, could be applied for mass proliferation and quality improvement of *E. alatus*.

Plant cell, tissue or organ culture normally requires sucrose as a carbon source for cell proliferation and development^30–32^. The availability of sucrose in the culture medium has been found to affect somatic embryogenesis in many woody plant species^33,34^. The effect of sucrose concentration on somatic embryo formation from embryogenic calli was examined in this study. The total number of somatic embryos was highest at 47.3 at 3% sucrose in this study. However, total number of somatic embryo decreased with increasing sucrose concentration (Fig. 3). These results are not consistent with previous reports that an increase in sucrose concentration promotes the development of embryogenic calli and somatic embryo^35,36^.

There are several contradictory reports on the role of light in the induction of somatic embryogenesis^37–39^. The total number of somatic embryos under dark incubation was slightly higher than that in light treatment (Fig. 3). However, the total number of somatic embryos from 7% (w/v) sucrose treatments was higher in the light than with dark incubation. These results clearly showed that light does not play a stimulatory role in early embryo development from embryogenic calli and when they incubated on culture medium containing below 5% sucrose of *E. alatus*.

Seed dormancy prevents seedling growth under unfavorable environmental conditions^40,41^. The germination of mature seeds of *E. alatus* is also delayed by prolonged seed dormancy^41^. Embryo growth is a critical step for the in vitro development and germination of somatic embryos. Upon transfer to MS medium supplemented with 0.1 mg/L BA or 1 mg/L GA_3 ,_the conversion frequencies of somatic embryos of *E. alatus* were 17% and 3%, respectively (Fig. 4). These results clearly show that BA treatment is more efficient than GA_3_ treatment for the conversion of somatic embryos. Our results are in good agreement with reports that cytokinins have been shown to be effective in plantlet conversion of somatic embryos from several plant species, including sorghum^42^, California poppy^43^, and *Schisandra chinensis*^22^.

In vitro-grown cell and tissue explants can secrete inhibitory phenolic compounds into the culture medium. Thus, adding AC to the media is very helpful in controlling browning and reducing polyphenol compounds during the whole culture period^44^. Additionally, AC absorbs 5-hydroxymethyl-2-furaldehide (HMF), a secondary product of autoclaving sucrose that reduces plant growth^45^. Furthermore, AC absorbs auxins liberated by the embryos into the medium during their development, which may interfere with embryo morphology and germination^46^. In this study, somatic embryos of *E. alatus* cultured on MS medium without AC germinated relatively poorly, but AC significantly improved their germination. Somatic embryos treated with AC successfully developed into healthy plantlets. The stimulatory effect of AC observed in this study was similar to previous reports showing a positive effect of AC on the conversion of somatic embryos into plantlets^47,48^. However, we did not observe a synergistic effect of BA and AC on the conversion of somatic embryos in this study.

To our knowledge, this is the first report of successful plant regeneration via somatic embryogenesis from cell suspension cultures. The cell suspension culture showed typical characteristics of embryogenic cells consisting of small isodiametric cell aggregates with dense cytoplasm. The embryogenic cell suspension cultures were repeatedly subcultured at 1-week intervals. After 4 weeks of incubation, cell aggregates from cell suspension cultures gave rise to numerous somatic embryos when transferred onto MS basal solid medium (Fig. 5c,d). Furthermore, embryogenic cell suspension cultures maintained embryogenic competence for more than 1 year. Plant cell suspension cultures have several advantages in the production of useful metabolites^49^ and recombinant proteins^14^. The biggest advantage is that plant cells can be easily proliferated for mass production under aseptic culture conditions. The seeds of *E. alatus* are excellent biological resources for the production of unusual acTAGs^3,4^. Currently, we are investigating the various physiological activities of crude extract from somatic embryos of *E. alatus.* In conclusion, an efficient plant regeneration system from embryogenic cell suspension cultures of *E. alatus* was established in this study. Therefore, the embryogenic cell line and plant regeneration system can be applied to genetic modification for pharmaceutical metabolite production from *E. alatus*.

## Materials and methods

### Plant materials and embryogenic callus formation from immature zygotic embryos

Immature zygotic embryos of *Euonymus alatus* (Thunb.) Sieb. were used for embryogenic callus induction. Immature seeds of were collected in the regional garden in Daejeon city, Republic of Korea in September 2019 and identified by Dr. Soo-Yong Kim at Korea Research Institute of Bioscience and Biotechnology (KRIBB). A voucher specimen (accession number KRIB 0030423) was preserved at the herbarium of the KRIBB. The use of plants or plant materials in the present study complies with international, national and/or institutional guidelines. Immature seeds were stored at 4 ℃ for 10 days before use.

Seeds were surface sterilized in 70% alcohol for 1 min, soaked in a sodium hypochlorite solution containing 0.8% active chloride for 20 min with occasional agitation and then washed five times with sterilized distilled water. The remaining moisture was removed with sterile filter papers (Advantec, 70 mm) in a laminar hood. After carefully removing the seed coats, immature zygotic embryos (approximately 2–3 mm in length) were transferred onto Murashige and Skoog^50^ (MS, 1962) inorganic salts containing 0.4 mg/L thiamine∙HCl, 100 mg/L myo-inositol, 30 g/L sucrose and 4 g/L Gelrite, pH 5.8 (adjusted with 1 N NaOH before autoclaving). All reagents related to plant tissue culture were purchased from Duchefa and plant growth regulators were purchased from Sigma.

To examine the effect of 2,4-dichlorophenoxyacetic acid (2,4-D) concentrations on embryogenic callus formation from immature embryos, immature embryos were placed onto MS medium supplemented with 0, 0.1, 0.5, 1, 2, and 5 mg/L 2,4-D. Each treatment consisted of 9 immature embryos in a Petri dish and was repeated three times. Unless otherwise stated, all cultures were maintained in a growth chamber at 25 °C in the dark. After 6 to 16 weeks of culture, we determined the frequency of white friable embryogenic callus formation from immature embryos.

To investigate whether the callus induced from immature embryos has embryogenic potential, white calli with nodule-like structures from radicle regions of zygotic embryos were transferred to MS basal medium without growth regulators. After 4 weeks of culture, the development of somatic embryos was examined.

### Effect of sucrose concentrations on somatic embryo formation from embryogenic calli

The effect of sucrose concentration on somatic embryo formation from embryogenic calli was also examined. Immature embryo-derived white embryogenic calli (approximately 2 mm in diameter) were transferred to MS medium containing 1 mg/L 2,4-D (MS1D) with 1, 3, 5, and 7% (w/v) sucrose. Each treatment consisted of 10 embryogenic calli in a Petri dish and was repeated three times. Furthermore, the effect of light and dark on somatic embryo formation was carried out in parallel with the sucrose treatments. For light incubation, the white embryogenic calli from immature embryo were cultured in a light incubator (approximately 40 ± 5 μmol m^−2^ s^−1^ from cool-white, fluorescent lamps with a 16-h photoperiod). After 5 weeks of culture, the total numbers of normal somatic embryos with two cotyledons out of various types somatic embryos were counted under a dissecting microscope (Nikon SMZ745, Japan).

### Whole plantlet regeneration from somatic embryos

To accelerate whole plant regeneration from somatic embryos, the effect of 6-benzyladenine (BA) and gibberellic acid (GA_3_) on further development of somatic embryos was examined. Somatic embryos of the torpedo stage were transferred to MS medium supplemented with 0.1 mg/L BA or 1 mg/L GA_3_. Furthermore, somatic embryos were transferred to MS medium supplemented with 0.2% (w/v) to investigate the effect of activated charcoal (Sigma-Aldrich) on the further development of somatic embryos. Each treatment consisted of 10 somatic embryos in a Petri dish and was repeated three times. After 4 weeks of culture in the light (approximately 40 ± 5 μmol m^−2^ s^−1^ from cool-white, fluorescent lamps with a 16-h photoperiod), the development of somatic embryos was examined.

### Establishment of embryogenic cell suspension cultures

Cell suspension cultures were initiated from immature zygotic embryo-derived white, friable, embryogenic calli. Approximately 1 g of white callus was transferred into an Erlenmeyer flask (250 mL) containing 20 mL of fresh MS1D liquid medium. The suspension cultures were maintained in a gyratory shaker (80 rpm) (Jeiotech IS-971RF, Korea) at 25 °C in the dark. After 1 week of incubation, an additional 10 mL of fresh MS1D liquid medium was added to the culture flask. After another-2 weeks of incubation, 20 mL fresh MS1D liquid medium was added to the flask. After the initial establishment of cell suspension cultures, 5 mL of cell suspension culture was transferred into an Erlenmeyer flask (250 mL) containing 50 mL of fresh MS1D liquid medium. This cell suspension culture was subcultured at 1-week intervals thereafter.

To check whether the cell aggregates from cell suspension cultures have the embryogenic potential, 4-week-old cell aggregates were transferred to MS basal medium without growth regulators. After 4 weeks of culture, the development of somatic embryos was examined.

### Statistical analysis

Data analyses were performed using the SPSS software, and the averages with the standard errors were compared by one-way ANOVA with the Duncan’s test (P < 0.05). Different letters in the figures indicate significant differences among the samples at a threshold of P < 0.05. Bars in all figures represent means ± SE determined from over three biological replicates. The number of experiments performed is indicated by the number n in the figure.

## Supplementary Information

Supplementary Information.

## Data Availability

All data generated or analyzed during this study are included in this published article and its Supplementary Information Files.
