# Supplementary material for: Efficient plant regeneration from embryogenic cell suspension cultures of Euonymus alatus
Source: Sci Rep. 2021 Jul 23;11:15120. doi: 10.1038/s41598-021-94597-4 (PMC8302629; doi:10.1038/s41598-021-94597-4)
Supplement: Supplementary file 1 — Supplementary Information. [file 41598_2021_94597_MOESM1_ESM.pdf]

Efficient plant regeneration from embryogenic cell suspension cultures of *Euonymus alatus*

Hyun A Woo[1,3], Seong Sub Ku[1], Eun Yee Jie[2], HyeRan Kim[1], Hyun-Soon Kim[1], Hye Sun Cho[1], Won-Joong Jeong[1], Sang Un Park[3], Sung Ran Min[1*], Suk Weon Kim[2*]

[1]Plant Systems Engineering Research Center, Korea Research Institute of Bioscience and Biotechnology, 125 Gwahak-ro, Yuseong-gu, Daejeon 34141, Republic of Korea.

[2]Biological Resource Center, Korea Research Institute of Bioscience and Biotechnology, 181 Ipsingil, Jeongeup-si, Jeollabuk-do 56212, Republic of Korea.

[3]Department of Crop Science, Chungnam National University, 99 Daehak-ro, Yuseong-gu, Daejeon 34134, Republic of Korea

Supplementary Figures

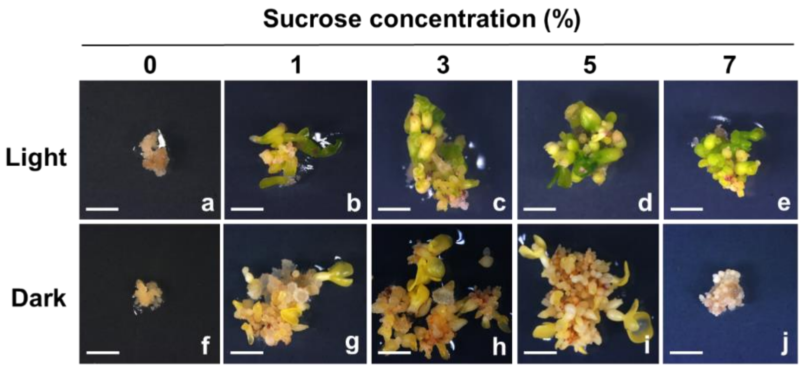

Supplementary Figure S1. Effects of sucrose concentrations and light requirement on embryogenic callus formation from immature embryos of *E. alatus*. The number of somatic embryos from different combinations of treatments of 0 (a and f), 1(b and g), 3 (c and h), 5(d and i), and 7% (e and j) sucrose in the light or dark incubation. Scale bars represent 2 mm.

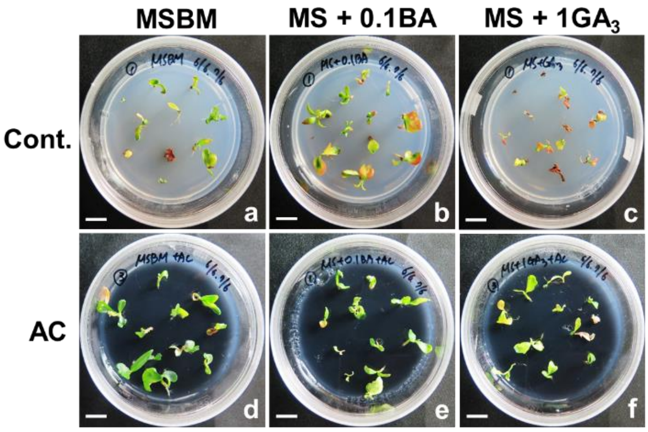

Supplementary Figure S2. Effects of growth regulators (BA and GA_3) and activated charcoal (AC) on the conversion of somatic embryos of *E. alatus* into plantlets. The conversion frequency of somatic embryos for different combinations of treatments of 0.1 mg/L BA, 1 mg/L GA_3 and 0.2% AC. Scale bars represent 1 cm.

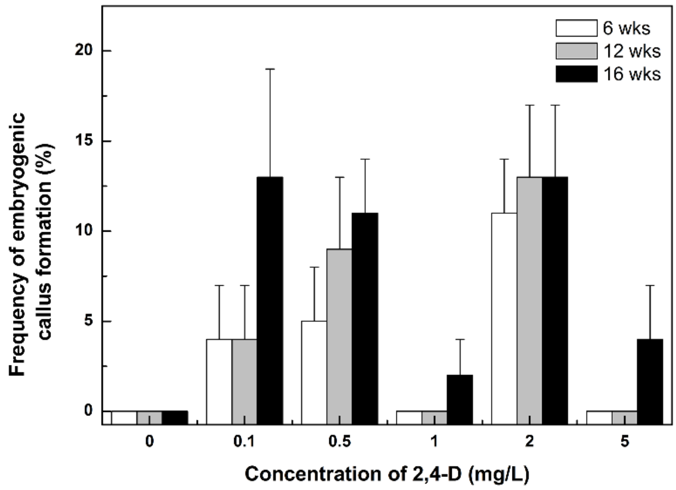

Supplementary Figure S3. Effects of 2,4-D concentrations on embryogenic callus formation from immature embryos from the seeds that did not undergo cold storage. Each symbol (-■-, -●-, -▲-) represent the incubation periods of immature embryos cultured on MS medium supplemented with several concentrations of 2,4-D. The frequency of embryogenic callus formation from different concentrations of 2,4-D treatments. Each treatment consisted of 9 immature zygotic embryos in a Petri dish and was repeated three times. Error bars represent SE (N = 27).
